# Discrimination Power of Polynomial-Based Descriptors for Graphs by Using Functional Matrices

**DOI:** 10.1371/journal.pone.0139265

**Published:** 2015-10-19

**Authors:** Matthias Dehmer, Frank Emmert-Streib, Yongtang Shi, Monica Stefu, Shailesh Tripathi

**Affiliations:** 1 Department of Computer Science, Universität der Bundeswehr München, Werner-Heisenberg-Weg 39, 85577 Neubiberg, Germany; 2 Department of Mechatronics and Biomedical Computer Science UMIT, Eduard Wallnoefer Zentrum 1, A-6060, Hall in Tyrol, Austria; 3 Computational Medicine and Statistical Learning Laboratory, Department of Signal Processing, Tampere University of Technology, Korkeakoulunkatu 1, FI-33720 Tampere, Finland; 4 Center for Combinatorics and LPMC-TJKLC, Nankai University, Tianjin, 300071, P.R. China; 5 School of Mathematics and Physics, Queen’s University Belfast, University Road, Belfast, BT7 1NN, United Kingdom; University of Maribor, SLOVENIA

## Abstract

In this paper, we study the discrimination power of graph measures that are based on graph-theoretical matrices. The paper generalizes the work of [M. Dehmer, M. Moosbrugger. Y. Shi, Encoding structural information uniquely with polynomial-based descriptors by employing the Randić matrix, Applied Mathematics and Computation, 268(2015), 164–168]. We demonstrate that by using the new functional matrix approach, exhaustively generated graphs can be discriminated more uniquely than shown in the mentioned previous work.

## Introduction

*Polynomial representations* have been investigated extensively in several application areas such as mathematical chemistry, discrete mathematics etc., see [1–5]. Some of these polynomials have been used as counting polynomials [2]. Another idea has been to define structural network measures based on the eigenvalues of graph polynomials, see [4, 6, 7]. A well-known example thereof is the *Hosoya index* [8] that has been defined by the coefficient of the so-called matching polynomial [3]. Another example relates to define spectral-based graph measures [9, 10]. Dehmer et al. [11] also made a contribution in this area by studying graph measures based on the zeros of the so-called *information polynomial*. It has been also Dehmer et al. [11] who explored the discrimination power of these polynomial-based measures. This relates to study the ability of these descriptors to distinguish non-isomorphic graphs structurally.

The paper is a successor of [6]. In [6], Dehmer et al. explored the discrimination of quantitative graph measures which are based on the eigenvalues of the Randić matrix. In this paper, we define a functional matrix that is more general than the Randić matrix. Therefore we expect an impact on the results when evaluating the discrimination power of the indices already used in [6] on exhaustively generated graphs. In fact, we find that by choosing a different structural setting than the one encoded by the Randić matrix, the proposed measures are even more unique than in [6].

## Methods and Results

### Novel Graph Measures Based on Complex Zeros

We use the idea of defining a matrix where its elements encode structural information as much as possible. For this, we use the information functional approach due to Dehmer [12]. An information functional is a function that maps the vertices to the reals; note that several information functionals have been already defined and they have been proven useful for discriminating graphs uniquely or classifying graphs efficiently, see [12, 13]. Let *G* = (*V*, *E*) a graph. As in [6, 14], we define the graph descriptors based on the zeros of

$$P_{M_{ij}^{g,f}}^{G}\left(z\right)\;≔\;\det\left(M_{ij}^{g,f}-zE\right),$$ (1)

where $M_{ij}^{g,f}$ is the *functional matrix* defined by

$$M_{ij}^{g,f}\;≔\;\{\begin{array}{cc} g(f\left(v_{j}\right),f\left(v_{j}\right)) & :\;(v_{i},v_{j})\in E\\ 0 & :\;\text{otherwise} \end{array}$$ (2)

As we can see, ${\left(M_{ij}^{g,f}\right)}_{ij}$ is based on using an information functional *f* and a function *g* composing *f*(*v*_*i*_) and *f*(*v*_*j*_), *v*_*i*_, *v*_*j*_ ∈ *V*. The composition function *g* should be symmetrical, so the eigenvalues of Eq 1 are then real-valued. It is clear that $P_{M_{ij}^{g,f}}^{G}\left(z\right)$ is given by

$$P_{M_{ij}^{g,f}}^{G}\left(z\right)=a_{n}z^{n}+a_{n-1}z^{n-1}+\cdots +a_{1}z+a_{0},\;a_{i}\in R.$$ (3)

We define the symmetric functions

$$g_{1}\left(f\left(v_{i}\right),f\left(v_{j}\right)\right)\;≔\;\frac{1}{\sqrt{f(v_{i})}\cdot \sqrt{f(v_{j})}},$$ (4)

$$g_{2}\left(f\left(v_{i}\right),f\left(v_{j}\right)\right)\;≔\;\sqrt{f(v_{i})}+\sqrt{f(v_{j})},$$ (5)

$$g_{3}\left(f\left(v_{i}\right),f\left(v_{j}\right)\right)\;≔\;\frac{1}{\sqrt{f(v_{i})}+\sqrt{f(v_{j})}}.$$ (6)

and the information functionals [13].

$$f^{1}\left(v_{i}\right)\;≔\;\sigma \left(v_{i}\right),$$ (7)

$$f^{2}\left(v_{i}\right)\;≔\;\delta \left(v_{i}\right),$$ (8)

and

$$\begin{array}{c} f^{3}(v_{i})\;≔\;c_{1}|S_{1}(v_{i},G)|+c_{2}|S_{2}(v_{i},G)|+\cdots +c_{\rho (G)}|S_{\rho (G)}(v_{i},G)|,\\ c_{k}>0,1\leq k\leq \rho (G),\alpha >0, \end{array}$$ (9)

*σ*(*v*_*i*_) is the eccentricity of a vertex *v*_*i*_ ∈ *V* [15], *δ*(*v*_*i*_) is the degree of *v*_*i*_ ∈ *V* [15], and *f* ^3^ is based in vertex spheres [12]; we have chosen the coefficients *c*_*k*_ linearly decreasing like

$$c_{1}\;≔\;\rho \left(G\right),\;c_{2}\;≔\;\rho \left(G\right)-1,\ldots ,\;c_{\rho (G)}\;≔\;1.$$ (10)

Note that in case of using the matrix $M_{ij}^{g_{1},f^{2}}$, we obtain the well-known Randić matrix [16]

$$R_{ij}\;≔\;\{\begin{array}{cc} {\left(\delta \left(v_{i}\right)\delta \left(v_{j}\right)\right)}^{-\frac{1}{2}} & :\;(v_{i},v_{j})\in E\\ 0 & :\;\text{otherwise} \end{array}$$ (11)

This matrix has already been used when evaluating the discrimination power of the graph measures representing Eqs 12–14, see [6]. Based on Eq 2, we see that *g*(*f*(*v*_*j*_), *f*(*v*_*j*_)) is taken into account and, hence, Eq 2 generalizes the Randić matrix. Therefore the here defined functional matrix enables us searching for cases where the functional matrix can encode structural information of graphs more uniquely than in case using the Randić matrix only, see [6].

Solving Eq 3, i.e., computing $P_{M_{ij}^{g,f}}^{G}\left(z\right)=0$, we determine the non-zero roots $R∍z_{1}^{M_{ij}^{g,f}},z_{2}^{M_{ij}^{g,f}},\ldots ,z_{k}^{M_{ij}^{g,f}}$.

In order to compare the new results with previous ones, we define as in [4] straightforwardly the following graph measures based on the zeros of ${\left(M_{ij}^{g,f}\right)}_{i,j}$:

$$M_{1}^{g,f}\left(G\right)\;≔\;|z_{1}^{M_{ij}^{g,f}}\left|+\right|z_{2}^{M_{ij}^{g,f}}\left|+\cdots +\right|z_{k}^{M_{ij}^{g,f}}|,$$ (12)

$$M_{2}^{g,f}\left(G\right)\;≔\;\sqrt{|z_{1}^{M_{ij}^{g,f}}|}+\sqrt{|z_{2}^{M_{ij}^{g,f}}|}+\cdots +\sqrt{|z_{k}^{M_{ij}^{g,f}}|},$$ (13)

and

$$\begin{array}{c} M_{3}^{g,f}\left(G\right)\;≔\;|z_{1}^{M_{ij}^{g,f}}\left|\cdot \log(\right|z_{1}^{M_{ij}^{g,f}}\left|)+\right|z_{2}^{M_{ij}^{g,f}}\left|\cdot \log(\right|z_{2}^{M_{ij}^{g,f}}\left|\right)\\ +\cdots +|z_{k}^{M_{ij}^{g,f}}\left|\cdot \log(\right|z_{k}^{M_{ij}^{g,f}}\left|\right). \end{array}$$ (14)

### Numerical Results

To interpret the numerical results, we start with giving some technical preliminaries. Here we describe how we generate exhaustively generated trees and graphs. We use the tree classes *T*_*i*_, 14 ≤ *i* ≤ 19 containing all non-isomorphic tress with *i* vertices. *N*_*i*_, 5 ≤ *i* ≤ 9 are the set of all non-isomorphic graphs with *i* vertices. The sizes of these classes are depicted in corresponding tables. As in [6], we generated the graphs by employing the package Nauty by McKay [17] and implemented the graph measures *M*_*i*_, 1 ≤ *i* ≤ 3 in R.

Now we start interpreting and discussing concrete numerical results; to do so, we start with trees. Tables 1–6 show the results when using *g* = *g*_1_ and *f* = *f* ^1^, *f* ^2^, *f* ^3^. We see that $M_{2}^{g,f}$ given by the sum of the square roots of the roots of $P_{M_{ij}^{g,f}}^{G}\left(z\right)=0$ is highly unique on exhaustively generated trees. In most of the cases, this measure is fully unique (ndv = 0 and *S* = 0). Also, it is evident that the case *f* = *f* ^2^, *g* = *g*_1_ and $M_{ij}^{g_{1},f^{2}}$ corresponds to the Randić matrix (see Tables 2 and 5 and [6]). We see that the cases *g* = *g*_1_ and *f* = *f* ^1^, *f* ^3^ give better results than in [6]. An explanation for this could be the better coverage of the local topological neighborhood of a vertex by the information functional *f* = *f* ^1^, *f* ^3^. In case of *f* ^3^, this seems very plausible as *f* ^3^ captures the full neighborhood of each vertex by using *j*-spheres [12]. In summary, all graph measures are highly unique on the chosen graph classes by using *g* = *g*_1_ and *f* = *f* ^1^, *f* ^2^, *f* ^3^. However the results are even more striking when considering Tables 7–18. We see that the measures $M_{1}^{g,f}-M_{3}^{g,f}$ are more unique when using *g* = *g*_2_ and *f* = *f* ^1^, *f* ^2^, *f* ^3^. It seems that *g* = *g*_2_ encodes/spreads its values more efficiently. This corresponds to an earlier finding due to Balaban et al. [18] where the descriptor $T=\sqrt{I_{1}}+\sqrt{I_{2}}+\cdots +\sqrt{I_{n}}$ was found to be highly unique; *I*_1_, …, *I*_*n*_ are topological indices. To study further details, see [18]. In this light, the zeros of the graph polynomial given by Eq 1 can be interpreted as structural graph descriptors (or topological indices). In particular, we observe the high uniqueness of $M_{1}^{g,f}-M_{3}^{g,f}$ when considering *g* = *g*_2_, *g* = *g*_3_ and the graph classes *T*_18_ and *T*_19_. Also note that *T*_19_ contains more than 300.000 connected graphs with 19 vertices each. Clearly, these results outperform the ones obtained in [6].

**Table 1:** Exhaustively generated sets of non-isomorphic trees. |*T*_14_| = 3159, |*T*_15_| = 7741, |*T*_16_| = 19320, |*T*_17_| = 48629. Here we used *f* = *f* ^1^, *g* = *g*_1_ and $M_{ij}^{g_{1},f^{1}}$.

	*T*_14_		*T*_15_		*T*_16_		*T*_17_	
Measure	ndv	*S*	ndv	*S*	ndv	*S*	ndv	*S*
$M_{1}^{g,f}$	6	0.9981	6	0.9992	12	0.9993	22	0.9995
$M_{2}^{g,f}$	0	1,0000	0	1,0000	0	1,0000	0	1,0000
$M_{3}^{g,f}$	0	1,0000	4	0.9994	8	0.9995	8	0.9998

**Table 2:** Exhaustively generated sets of non-isomorphic trees. |*T*_14_| = 3159, |*T*_15_| = 7741, |*T*_16_| = 19320, |*T*_17_| = 48629. Here we used *f* = *f* ^2^, *g* = *g*_1_ and $M_{ij}^{g_{1},f^{2}}$.

	*T*_14_		*T*_15_		*T*_16_		*T*_17_	
Measure	ndv	*S*	ndv	*S*	ndv	*S*	ndv	*S*
$M_{1}^{g,f}$	30	0,9905	62	0,9919	126	0,9934	228	0,9953
$M_{2}^{g,f}$	0	1,0000	0	1,0000	2	0,9998	0	1,0000
$M_{3}^{g,f}$	2	0,9993	4	0,9994	8	0,9995	8	0,9998

**Table 3:** Exhaustively generated sets of non-isomorphic trees. |*T*_14_| = 3159, |*T*_15_| = 7741, |*T*_16_| = 19320, |*T*_17_| = 48629. Here we used *f* = *f* ^3^, *g* = *g*_1_ and $M_{ij}^{g_{1},f^{3}}$.

	*T*_14_		*T*_15_		*T*_16_		*T*_17_	
Measure	ndv	*S*	ndv	*S*	ndv	*S*	ndv	*S*
$M_{1}^{g,f}$	0	1.0000	0	1.0000	0	1.0000	0	1.0000
$M_{2}^{g,f}$	0	1.0000	0	1.0000	0	1.0000	0	1.0000
$M_{3}^{g,f}$	0	1.0000	0	1.0000	0	1.0000	0	1.0000

**Table 4:** Exhaustively generated sets of non-isomorphic trees. |*T*_18_| = 123867, |*T*_19_| = 317955. Here we used *f* = *f* ^1^, *g* = *g*_1_ and $M_{ij}^{g_{1},f^{1}}$.

	*T*_18_		*T*_19_	
Measure	ndv	*S*	ndv	*S*
$M_{1}^{g,f}$	42	0.9996	68	0.9997
$M_{2}^{g,f}$	0	1,0000	0	1,0000
$M_{3}^{g,f}$	10	0.9999	24	0.9999

**Table 5:** Exhaustively generated sets of non-isomorphic trees. |*T*_18_| = 123867, |*T*_19_| = 317955. Here we used *f* = *f* ^2^, *g* = *g*_1_ and $M_{ij}^{g_{1},f^{2}}$.

	*T*_18_		*T*_19_	
Measure	ndv	*S*	ndv	*S*
$M_{1}^{g,f}$	528	0,9957	693	0,9978
$M_{2}^{g,f}$	8	0,9999	0	1,0000
$M_{3}^{g,f}$	14	0,9998	40	0,9998742

**Table 6:** Exhaustively generated sets of non-isomorphic trees. |*T*_18_| = 123867, |*T*_19_| = 317955. Here we used *f* = *f* ^3^, *g* = *g*_1_ and $M_{ij}^{g_{1},f^{3}}$.

	*T*_18_		*T*_19_	
Measure	ndv	*S*	ndv	*S*
$M_{1}^{g,f}$	0	1.0000	0	1.0000
$M_{2}^{g,f}$	0	1.0000	0	1.0000
$M_{3}^{g,f}$	0	1.0000	0	1.0000

**Table 7:** Exhaustively generated sets of non-isomorphic trees. |*T*_14_| = 3159, |*T*_15_| = 7741, |*T*_16_| = 19320, |*T*_17_| = 48629. Here we used *f* = *f* ^1^, *g* = *g*_2_ and $M_{ij}^{g_{2},f^{1}}$.

	*T*_14_		*T*_15_		*T*_16_		*T*_17_	
Measure	ndv	*S*	ndv	*S*	ndv	*S*	ndv	*S*
$M_{1}^{g,f}$	0	1.0000	0	1.0000	0	1.0000	0	1.0000
$M_{2}^{g,f}$	0	1.0000	0	1.0000	0	1.0000	0	1.0000
$M_{3}^{g,f}$	0	1.0000	0	1.0000	0	1.0000	0	1.0000

**Table 8:** Exhaustively generated sets of non-isomorphic trees. |*T*_14_| = 3159, |*T*_15_| = 7741, |*T*_16_| = 19320, |*T*_17_| = 48629. Here we used *f* = *f* ^2^, *g* = *g*_2_ and $M_{ij}^{g_{2},f^{2}}$.

	*T*_14_		*T*_15_		*T*_16_		*T*_17_	
Measure	ndv	*S*	ndv	*S*	ndv	*S*	ndv	*S*
$M_{1}^{g,f}$	0	1.0000	0	1.0000	0	1.0000	0	1.0000
$M_{2}^{g,f}$	0	1.0000	0	1.0000	0	1.0000	0	1.0000
$M_{3}^{g,f}$	0	1.0000	0	1.0000	0	1.0000	0	1.0000

**Table 9:** Exhaustively generated sets of non-isomorphic trees. |*T*_14_| = 3159, |*T*_15_| = 7741, |*T*_16_| = 19320, |*T*_17_| = 48629. Here we used *f* = *f* ^3^, *g* = *g*_3_ and $M_{ij}^{g_{3},f^{3}}$.

	*T*_14_		*T*_15_		*T*_16_		*T*_17_	
Measure	ndv	*S*	ndv	*S*	ndv	*S*	ndv	*S*
$M_{1}^{g,f}$	0	1.0000	0	1.0000	0	1.0000	0	1.0000
$M_{2}^{g,f}$	0	1.0000	0	1.0000	0	1.0000	0	1.0000
$M_{3}^{g,f}$	0	1.0000	0	1.0000	0	1.0000	0	1.0000

**Table 10:** Exhaustively generated sets of non-isomorphic trees. |*T*_18_| = 123867, |*T*_19_| = 317955. Here we used *f* = *f* ^1^, *g* = *g*_2_ and $M_{ij}^{g_{2},f^{1}}$.

	*T*_18_		*T*_19_	
Measure	ndv	*S*	ndv	*S*
$M_{1}^{g,f}$	4	0.9987	2	0.9993
$M_{2}^{g,f}$	0	1.0000	0	1.0000
$M_{3}^{g,f}$	0	1.0000	0	1.0000

**Table 11:** Exhaustively generated sets of non-isomorphic trees. |*T*_18_| = 123867, |*T*_19_| = 317955. Here we used *f* = *f* ^2^, *g* = *g*_2_ and $M_{ij}^{g_{2},f^{2}}$.

	*T*_18_		*T*_19_	
Measure	ndv	*S*	ndv	*S*
$M_{1}^{g,f}$	0	1.0000	0	1.0000
$M_{2}^{g,f}$	0	1.0000	0	1.0000
$M_{3}^{g,f}$	0	1.0000000	0	1.0000

**Table 12:** Exhaustively generated sets of non-isomorphic trees. |*T*_18_| = 123867, |*T*_19_| = 317955. Here we used *f* = *f* ^3^, *g* = *g*_2_ and $M_{ij}^{g_{2},f^{3}}$.

	*T*_18_		*T*_19_	
Measure	ndv	*S*	ndv	*S*
$M_{1}^{g,f}$	0	1.0000	0	1.0000
$M_{2}^{g,f}$	0	1.0000	0	1.0000
$M_{3}^{g,f}$	0	1.0000	0	1.0000

**Table 13:** Exhaustively generated sets of non-isomorphic trees. |*T*_14_| = 3159, |*T*_15_| = 7741, |*T*_16_| = 19320, |*T*_17_| = 48629. Here we used *f* = *f* ^1^, *g* = *g*_3_ and $M_{ij}^{g_{3},f^{1}}$.

	*T*_14_		*T*_15_		*T*_16_		*T*_17_	
Measure	ndv	*S*	ndv	*S*	ndv	*S*	ndv	*S*
$M_{1}^{g,f}$	0	1.0000	0	1.0000	0	1.0000	0	1.0000
$M_{2}^{g,f}$	0	1.0000	0	1.0000	0	1.0000	0	1.0000
$M_{3}^{g,f}$	0	1.0000	0	1.0000	0	1.0000	0	1.0000

**Table 14:** Exhaustively generated sets of non-isomorphic trees. |*T*_14_| = 3159, |*T*_15_| = 7741, |*T*_16_| = 19320, |*T*_17_| = 48629. Here we used *f* = *f* ^2^, *g* = *g*_3_ and $M_{ij}^{g_{3},f^{2}}$.

	*T*_14_		*T*_15_		*T*_16_		*T*_17_	
Measure	ndv	*S*	ndv	*S*	ndv	*S*	ndv	*S*
$M_{1}^{g,f}$	0	1.0000	0	1.0000	0	1.0000	2	0.9993
$M_{2}^{g,f}$	0	1.0000	0	1.0000	0	1.0000	0	1.0000
$M_{3}^{g,f}$	0	1.0000	0	1.0000	0	1.0000	0	1.0000

**Table 15:** Exhaustively generated sets of non-isomorphic trees. |*T*_14_| = 3159, |*T*_15_| = 7741, |*T*_16_| = 19320, |*T*_17_| = 48629. Here we used *f* = *f* ^3^, *g* = *g*_3_ and $M_{ij}^{g_{3},f^{3}}$.

	*T*_14_		*T*_15_		*T*_16_		*T*_17_	
Measure	ndv	*S*	ndv	*S*	ndv	*S*	ndv	*S*
$M_{1}^{g,f}$	0	1.000000	0	1.000	0	1.0000	0	1.0000
$M_{2}^{g,f}$	0	1.0000	0	1.0000	0	1.0000	0	1.0000
$M_{3}^{g,f}$	0	1.0000	0	1.0000	0	1.0000	0	1.0000

**Table 16:** Exhaustively generated sets of non-isomorphic trees. |*T*_18_| = 123867, |*T*_19_| = 317955. Here we used *f* = *f* ^1^, *g* = *g*_3_ and $M_{ij}^{g_{3},f^{1}}$.

	*T*_18_		*T*_19_	
Measure	ndv	*S*	ndv	*S*
$M_{1}^{g,f}$	0	1.0000	0	1.0000
$M_{2}^{g,f}$	0	1.0000	0	1.0000
$M_{3}^{g,f}$	0	1.0000	0	1.0000

**Table 17:** Exhaustively generated sets of non-isomorphic trees. |*T*_18_| = 123867, |*T*_19_| = 317955. Here we used *f* = *f* ^2^, *g* = *g*_3_ and $M_{ij}^{g_{3},f^{2}}$.

	*T*_18_		*T*_19_	
Measure	ndv	*S*	ndv	*S*
$M_{1}^{g,f}$	10	0.9968	24	0.9924
$M_{2}^{g,f}$	0	1.0000	0	1.0000
$M_{3}^{g,f}$	2	0.9993	2	0.9993

**Table 18:** Exhaustively generated sets of non-isomorphic trees. |*T*_18_| = 123867, |*T*_19_| = 317955. Here we used *f* = *f* ^3^, *g* = *g*_3_ and $M_{ij}^{g_{3},f^{3}}$.

	*T*_18_		*T*_19_	
Measure	ndv	*S*	ndv	*S*
$M_{1}^{g,f}$	0	1.0000	0	1.0000
$M_{2}^{g,f}$	0	1.0000	0	1.0000
$M_{3}^{g,f}$	0	1.0000	0	1.0000

As to the graphs, we look at Tables 19–21. We see that the results are shown for *g* = *g*_1_ and *f* = *f* ^1^, *f* ^2^, *f* ^3^. The results for *g* = *g*_2_ and *g* = *g*_3_ are very similar and therefore they are not shown. We emphasize that most of the obtained results are much better than the ones in [6]. In this paper, the lowest ndv-value for *N*_9_ achieved by using $M_{2}^{g,f}$ equals 8 (see Table 20); that means 99.9996% out of 261080 graphs could be discriminated uniquely. This is a striking result and outperforms all earlier results done by Dehmer et al. and co-workers, see, [4, 6, 19–22].

**Table 19:** Exhaustively generated sets of non-isomorphic graphs. ∣*N*_6_∣ = 112, ∣*N*_7_∣ = 853, ∣*N*_8_∣ = 11117, ∣*N*_9_∣ = 261080. Here we used *f* = *f* ^1^, *g* = *g*_1_ and $M_{ij}^{g_{1},f^{1}}$.

	*N*_5_		*N*_6_		*N*_7_		*N*_8_		*N*_9_	
Measure	ndv	*S*	ndv	*S*	ndv	*S*	ndv	*S*	ndv	*S*
$M_{1}^{g,f}$	0	1.0000	5	0.9553	18	0.9788	408	0.9633	13305	0.9490
$M_{2}^{g,f}$	0	1.0000	0	1.0000	2	0.9976	181	0.9837	6668	0.9744
$M_{3}^{g,f}$	0	1.0000	0	1.0000	4	0.9953	112	0.9899	6392	0.9755

**Table 20:** Exhaustively generated sets of non-isomorphic graphs. ∣*N*_6_∣ = 112, ∣*N*_7_∣ = 853, ∣*N*_8_∣ = 11117, ∣*N*_9_∣ = 261080. Here we used *f* = *f* ^2^, *g* = *g*_1_ and $M_{ij}^{g_{1},f^{2}}$.

	*N*_5_		*N*_6_		*N*_7_		*N*_8_		*N*_9_	
Measure	ndv	*S*	ndv	*S*	ndv	*S*	ndv	*S*	ndv	*S*
$M_{1}^{g,f}$	6	0.7142	13	0.8839	33	0.9613	71	0.9936	272	0.9989
$M_{2}^{g,f}$	0	1.0000	0	1.0000	4	0.9953	13	0.9988	103	0.9996
$M_{3}^{g,f}$	2	0.9047	0	1.0000	8	0.9906	23	0.9979	183	0.9992

**Table 21:** Exhaustively generated sets of non-isomorphic graphs. ∣*N*_6_∣ = 112, ∣*N*_7_∣ = 853, ∣*N*_8_∣ = 11117, ∣*N*_9_∣ = 261080. Here we used *f* = *f* ^3^, *g* = *g*_1_ and $M_{ij}^{g_{1},f^{3}}$.

	*N*_5_		*N*_6_		*N*_7_		*N*_8_		*N*_9_	
Measure	ndv	*S*	ndv	*S*	ndv	*S*	ndv	*S*	ndv	*S*
$M_{1}^{g,f}$	0	1.0000	2	0.9821	0	1.0000	4	0.9996	20	0.9999
$M_{2}^{g,f}$	0	1.0000	0	1.0000	0	1.0000	2	0.9998	8	0.9999
$M_{3}^{g,f}$	0	1.0000	0	1.0000	0	1.0000	4	0.9996	18	0.9999

In comparison, the lowest ndv-value for *N*_9_ equals 126 which has been achieved in [6] by using the entropy-like measure $M_{3}^{g,f}$. Finally, we see that the results we have obtained in this paper are in parts slightly better and outperform the previous approach by using the Randić matrix.

## Conclusion

In this paper, we generalized the work done by Dehmer et al. [6]. In [6], the eigenvalues of the Randić matrix have been used to define new quantitative network measures. A result of this work [6] was the high discrimination power of the measures on exhaustively generated networks. Note that the definition of Randić matrix comes from the famous Randić index [23, 24], from which some new invariants have been introduced, such as the Randić spectral [25] and the Randić energy [26].

Based on the construction of the functional matrix (see Eq 2), we have expected that the here proposed approach may be useful to discriminate graphs more efficiently than by using earlier methods, see, e.g., [4, 6]. From a mathematical point of view, this seems plausible as the involved information functionals *f* ^1^, …, *f* ^3^ encode structural information differently. In particular, *f* ^3^ captures the full topological neighborhood of a vertex and, hence, it encodes structural information more efficiently than *f* ^1^. This effect can be seen by the Tables 1–21. On top of that, the composite functions *g*_1_, …, *g*_3_ may optimize the spread of values of the involved information functionals. We therefore conclude that the ability how the final graph measure can discriminate graphs structurally also depends on the composite function *g*. In this case, evidence of this statement follows as we defined settings different from the Randić matrix, i.e., *f* = *f* ^2^, *g* = *g*_1_ and finally $M_{ij}^{g_{1},f^{2}}$.
